# Characterization of the complete mitochondrial genome of *Alectryonella plicatula* (Bivalvia: Ostreidae)

**DOI:** 10.1080/23802359.2021.1915205

**Published:** 2021-05-04

**Authors:** Qingzhi Wang, Hongyue Liu, Weiming Teng, Zuoan Yu, Xiangfeng Liu, Xi Xie, Chao Yue, Dacheng Li, Miao Liang, Qi Li

**Affiliations:** a Key Laboratory of Mariculture, Ministry of Education, Ocean University of China, Qingdao, China; b Liaoning Ocean and Fisheries Science Research Institute and Dalian Key Laboratory of Genetic Resources for Marine Shellfish, Dalian, China; c National Marine Environmental Monitoring Center, Dalian, China

**Keywords:** *Alectryonella plicatula*, mitogenome, grass oyster

## Abstract

In this study, we sequenced and analyzed the complete mitochondrial genome of *Alectryonella plicatula* (Gmelin 1791), the newly determined mitochondrial genome is 18225 bp in length, it is a circular molecule and consists of 12 protein-coding genes (*atp8* is absent), 24 transfer RNA (with two copies of *trnP* and *trnQ*), and 2 ribosomal RNA genes (splitting of the *rrnL* gene and duplication of the *rrnS* gene were identified). Phylogenetic analysis based on 12 protein coding genes showed that *Alectryonella plicatula* is closely related to *Crassostrea gigas*.

*Alectryonella plicatula* is a native species that distributed along the coast of China, commonly known as ‘grass oyster’, ‘zhe oyster’. It mainly inhabiting intertidal hard grounds can tolerate a wide range of salinity and temperature (Wang 1988). The shell of *A. plicatula* is relatively thin and often smaller than other cupped oyster *Crassostrea* species (Yu et al. 2008). To date, studies use molecular tools to understand the taxonomic status of *A. plicatula* and discriminate this species from others have only used partial genomes ( Liu and Dai 1998; Yu et al. 2008, Wang et al. 2008). The complete mitochondrial genome of *A. plicatula* is thus of great importance to shed some light on the diversity and phylogenetic relationship of Ostreidae.

In this study, the complete mitochondrial genome of *A. plicatula* was determined by next-generation sequencing (NGS) platform. Specimen was collected from Shicheng Island, Dalian (122° 98′ 38E, 39° 56′ 69 N) and stored at Liaoning Ocean and Fisheries Science Research Institute, Dalian, China (voucher no. CP-202005). After collection, specimens were immediately preserved in 95% ethanol until DNA extraction. Total genomic DNA was isolated using the DNeasy tissue kit (Qiagen) according to the manufacturer’s instructions. Total genomic DNA was sequenced on an Illumina HiSeq sequencer using a PE150 protocol. The raw reads were filtered using Trimmomatics (Bolger et al. 2014), and were assembled using NOVOPlasty (Dierckxsens et al. 2017).

The complete mitochondrial genome of *A. plicatula* (Genbank assession number MW143047) is 18225 bp in length, according to the annotation results from MITOS (Bernt et al. 2013) and ORF Finder (https://www.ncbi.nlm.nih.gov/orffinder/), it contains 12 protein-coding genes (as in most bivalves, the *atp8* gene is missing), 24 tRNA genes, 2 rRNA genes, and the splitting of the *rrnL* gene and duplication of the *rrnS* gene were identified. Typical metazoan mitogenome have a set of 22 tRNA genes, including two copies of *trnL* and two of *trnS* (Podsiadlowski et al. 2008), but the number of tRNA genes in oyster mitogenomes varies (Wu et al. 2010). The mitogenome of *A. plicatula* also did not follow that rule, which include extra copies of *trnM* and *trnQ*. The nucleotide composition of this mitogenome is A 27.6%, C 14.6%, G 22.0%, T 35.7%, with A + T content (63.3%) higher than G + C content (36.7%), the nucleotide compositions and AT contents of the entire mitogenome is similar to the pattern of other oyster mitogenomes (Wu et al. 2010).

The phylogenetic relationships of *A. plicatula* to *Crassostrea* species were reconstructed based on the 12 protein coding genes using maximum-likelihood analysis, three members from *Ostrea* (Bivalvia: Ostreidae) were selected to root the tree (Figure 1). *A. plicatula* was recovered as the sister group of *C. gigas* with the maximal support, thus validated the close relationship between the two species. This clade is then group together with *C. angulate*, and *C. sikamea* is the sister to the clade containing *C. angulate* and *C. gigas* + *A. plicatula*.

**Figure 1.**
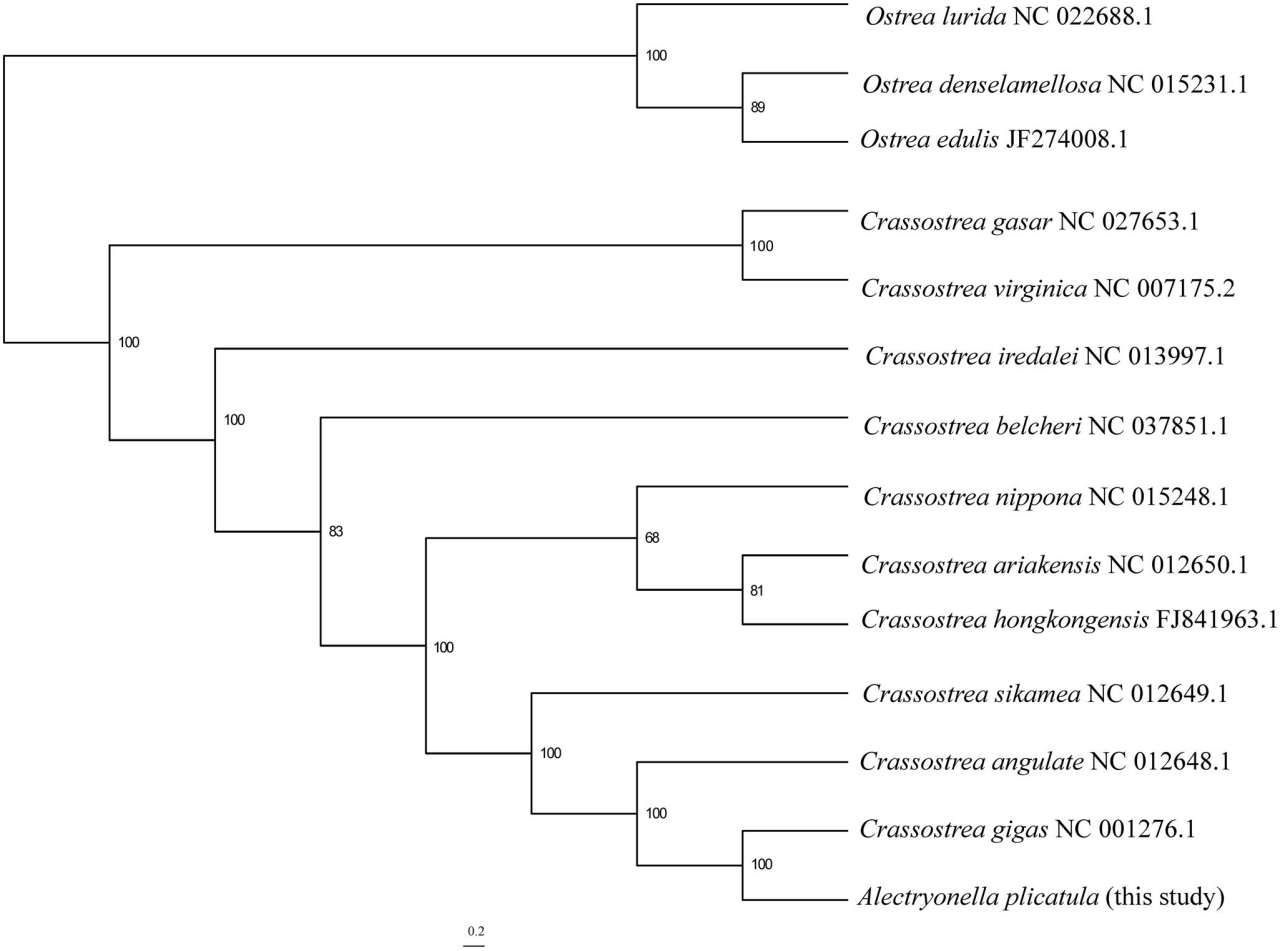
The maximum-likelihood (ML) phylogenetic relationships of *Crassostrea* based on the 12 protein coding genes using IQ-TREE v1.6.1(Nguyen et al. 2015). The ML searches were run using a combination of rapid hill-climbing approaches and the stochastic perturbation method with 1,000 ultrafast bootstraps.

The complete mitogenome of *A. plicatula* presents here provide valuable resources for accurate molecular barcoding and identification of specimens of this native species. In addition, it will also be useful for investigating the phylogeny and population genetics of Asian oyster species.

## Data Availability

The data that support the findings of this study are openly available in NCBI at https://www.ncbi.nlm.nih.gov under the accession number MW143047, or available from the corresponding author. The raw sequence data were deposited in SRA, accession number SRR13449415.
